# Identification of Parkinson’s Disease-Causing Genes via Omics Data

**DOI:** 10.3389/fgene.2021.712164

**Published:** 2021-07-28

**Authors:** Xinran Cui, Chen Xu, Liyuan Zhang, Yadong Wang

**Affiliations:** Center for Bioinformatics, School of Computer Science and Technology, Harbin Institute of Technology, Harbin, China

**Keywords:** Parkinson’s disease, SMR analysis, GWAS summary data, eQTL summary data, risk genes

## Abstract

Parkinson’s disease (PD) is the second most frequent neurogenic disease after Alzheimer’s disease. The clinical manifestations include mostly motor disorders, such as bradykinesia, myotonia, and static tremors. Since the cause of this pathological features remain unclear, there is currently no radical treatment for PD. Environmental and genetic factors are thought to contribute to the pathology of PD. To identify the genetic factors, some studies employed the Genome-Wide Association Studies (GWAS) method and detected certain genes closely related to PD. However, the functions of these gene mutants in the development of PD are unknown. Combining GWAS and expression Quantitative Trait Loci (eQTL) analysis, the biological meaning of mutation could be explained to some extent. Therefore, the present investigation used Summary data-based Mendelian Randomization (SMR) analysis to integrate of two PD GWAS datasets and four eQTL datasets with the objective of identifying casual genes. Using this strategy, we found six Single Nucleotide Polymorphism (SNP) loci which could cause the development of PD through altering the susceptibility gene expression, and three risk genes: Synuclein Alpha (SNCA), Mitochondrial Poly(A) Polymerase (MTPAP), and RP11-305E6.4. We proved the accuracy of results through case studies and inferred the functions of these genes in PD. Overall, this study provides insights into the genetic mechanism behind PD, which is crucial for the study of the development of this disease and its diagnosis and treatment.

## Introduction

Parkinson’s disease (PD) is the second most common degenerative disorder of the nervous system. As the incidence of this disease is strongly linked to age, approximately 1% of 65-year-olds has this disease, rising to 4–5% among aged 85 (Trinh and Farrer, 2013). Statistically, the rate of PD increases 5–10 times from the age of 60 to the age of 90 (Poewe et al., 2017). The main clinical manifestations are involuntary limb tremor, bradykinesia, walking difficulty, and stiff limbs, and these symptoms become aggravated with time. This causes that PD patients are peculiarly prone to falls on routine activities. The incidence of falls could reach 40–70% (Kerr et al., 2010). Falling can lead to injury and the decrease of survival for PD patients. Thus, the health and life of human beings, especially the elderly, are threatened by this disease.

There are two main pathologic characteristics of PD. One is the degeneration and death of dopamine neurons in the substantia nigra pars compacta of the midbrain and the consequent depletion of dopamine in the striatum (Ammal Kaidery and Thomas, 2018). Dopamine is synthesized by dopamine neurons, and then delivered to the striatum to regulate somatic motor (Schwarz and Peever, 2011). The other feature is the formation of acidophilic inclusions known as Lewy bodies in the cytoplasm of the remaining neurons in the substantia nigra (Feng et al., 2020). Although many studies have explored the pathological mechanism of PD, there is still no sufficient evidence to explain the degeneration of dopaminergic neurons and the formation of Lewy bodies, thus the current PD treatment approaches can only relieve symptoms with medication but cannot reverse this disease progression (Ball et al., 2019). Moreover, long-term treatment results in the development of drug resistance, and the available drugs have significant adverse effects. Therefore, it is urgent to understand the biological process leading to these pathological changes for the cure of PD.

Some studies revealed that both environmental and genetic factors contribute to the pathological features of PD. The main biological mechanism by which environmental factors can damage dopaminergic neurons involves the inhibition of the activity of mitochondrial complex enzymes and the mitochondrial respiratory chain (Holper et al., 2019). In addition to environmental factors, some patients may have inherited certain particular mutated gene that lead to the development of PD (Antony et al., 2013). Understanding what these genes do has crucial implications for understanding the changes in the biological processes underlying PD. Thus, it is necessary to reveal these mutant genes for conquering this disease.

A genome-wide association study (GWAS) identified more than 10 PD-causing genes (Nalls et al., 2014). GWAS is a strategy for identifying common genetic variants [Single Nucleotide Polymorphism (SNP)] significantly associated with a complex trait or a disease in the whole human genome, thus recognizing the disease-related genes (Cannon and Mohlke, 2018). In the results of GWAS analysis, most significant SNP sites were located in non-coding regions, making it difficult to directly explore the regulatory mechanism of these sites (Rojano et al., 2016). Thus, there are some PD risk genes revealed in many GWAS studies, what role do these genes play in the development of PD remains unknown.

The combined analysis of GWAS and expression Quantitative Trait Loci (eQTL) has become an important means to reveal the function of significant variants. Although GWAS have identified thousands of variants associated with complex traits, their biological explanation is often still unclear. Most of these variants overlap with eQTL, suggesting that they may be involved in the regulation of gene expression (Zhu et al., 2016). Genes associated with these variants could be regarded as PD causing-genes. This study exploited Summary data-based Mendelian Randomization analysis (SMR) to integrate and analyze the PD summary data of the GWAS with the summary data of eQTL, to explore the genetic mechanism by which certain disease-causing genes contribute to PD. The SMR analysis method does not require the data with both genotype and gene expression and a massive size. Thus, this approach could leverage the published data to a large extent. The statistical analysis of the relationship between a single SNP and gene expression is called the eQTL analysis (Shabalin, 2012). If the expression of a gene is affected by a SNP, then this genetic variant is considered as an eQTL locus. Since SNP is the subject of study in both GWAS and eQTL, SNP is used as an instrumental variable in the SMR method to determine which genes expression changes could lead to the occurrence of PD. Thus, the SMR analysis results may provide a direction for the treatment of PD.

## Materials and Methods

### Data Acquisition

Two public summary datasets of GWAS for PD were downloaded from the GWAS catalog website. One of the GWAS datasets represented data from 282,871 white British inpatient samples reported by the UK Biobank. The UK Biobank is a cohort study collecting, physical, and health data of approximately 500,000 British individuals. For the purpose of this analysis, this dataset was named “GUB” (Bi et al., 2020). The other GWAS dataset is based on the genetic data of 28,568 PD patients obtained from International Parkinson’s Disease Genomics Consortium and was named “GIPD” (Blauwendraat et al., 2019). This data consists of Parkinson’s patients from European countries such as United Kingdom, Dutch, Finnish, and German. The present study also employed four eQTL datasets. EQTL data are generally collected from peripheral blood, thus one dataset is the summary level statistics of eQTL from the Consortium for the Architecture of Gene Expression (CAGE) data. It provides the measurements of the level of gene expression in peripheral blood. This dataset contains more than 3 million SNPs, identified by 33,323 probes. Since PD is a neurodegenerative disease, the second dataset includes the level of gene expression in brain tissue and includes information on 28,522 probes and more than 13 million SNPs. To explore whether PD is associated with other factors such as reduced immunity, we selected two sets of eQTL data sets for T cells. The remaining two datasets list the level of gene expression in CD4- and CD8-positive cells, respectively. CD4 and CD8 are both markers of T lymphocytes. The CD4 dataset includes more than five hundred thousand SNPs, measured by 7,350 probes, while the CD8 dataset includes more than three hundred thousand SNPs through using 5,829 probes.

### SMR Analysis

Both GWAS and eQTL were used to investigate the relationship between SNP and traits or gene expression through linear regression analysis. In the regression analysis, the effect size (beta-value) corresponds to the value of the regression coefficient, while SE stands for the standard error of the regression coefficient. Then, the GWAS and eQTL data were standardized using the Z-score method, in which the Z was calculated as the quotient of the beta-value and the SE-value.

**FIGURE 1:**
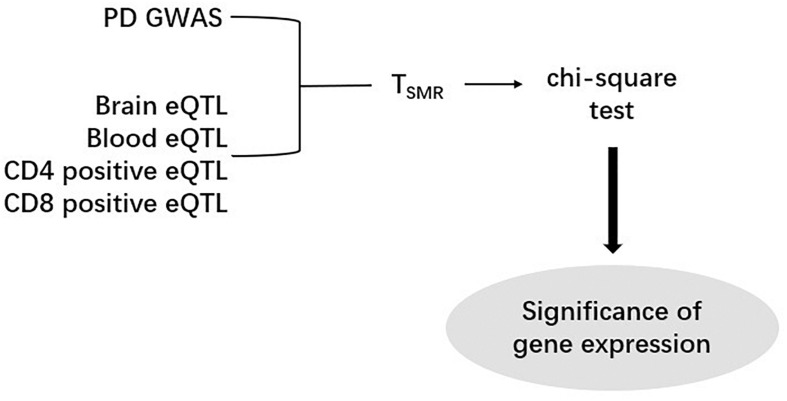
The Summary data-based Mendelian Randomization (SMR) analysis flow chart. One PD GWAS was integrated with each of eQTL data. After calculating T_*SMR*_ and chi-square test, the significance of gene expression were discovered.

After computing the Z-score, we performed SMR analysis on an eQTL dataset and a GWAS dataset (Figure 1). Since SNPs are regarded as instrumental variables in SMR analysis, we identified the overlapped SNPs between an eQTL dataset and a GWAS dataset and then generated a new dataset including all eQTL and GWAS data of the overlapped SNPs. As a result, eight new datasets were obtained by this approach, and each of them was subsequently subjected to the SMR analysis. According to the formula,

TSMR≈ZGWAS2×ZeQTL2ZGWAS2+ZeQTL2

the Z values of GWAS and eQTL were used to calculate the T_*SMR*_ value. The chi-square test was applied to the T_*SMR*_ values to calculate their *P* values (P_*SMR*_). Each SMR dataset has a specific threshold. This threshold is calculated by dividing 0.05 by the number of probes in the corresponding eQTL dataset. If we find that some P_*SMR*_ values are less than the threshold value of their data set, the genes corresponding to these P_*SMR*_ can be considered as risk genes.

## Results

### Identification of Overlapping SNPs

Since SNP is an instrumental variable, we searched for the same SNPs between a GWAS dataset and an eQTL dataset, generating eight new datasets (Table 1 and Supplementary Figures 1, 2). The results showed that both GUB_Brain and GIPD_Brain datasets contain more than 10 million overlapped SNPs and about 28, 000 genes. There are over two million SNPs and about 20,000 in GUB_Cage and the GIPD_Cage datasets. The other four data have fewer than half a million SNPs and about four to five thousand genes.

**TABLE 1:** The new dataset list for SMR analysis.

eQTL	GWAS			
	Brain	Cage	CD4	CD8
GUB	GUB_Brain	GUB_Cage	GUB_CD4	GUB_CD8
GIPD	GIPD_Brain	GIPD_Cage	GIPD_CD4	GIPD_CD8

*Each GWAS data combined with each eQTL data were analyzed to produce a new SMR data. Thus, there were eight SMR datasets.*

**FIGURE 2:**
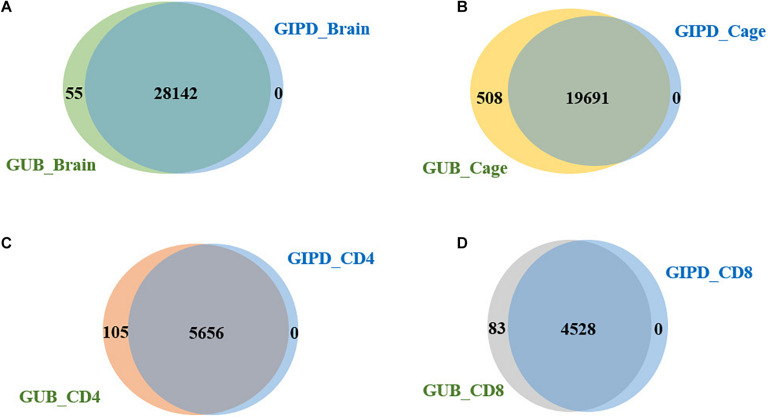
**(A)** The overlap genes of the GUB_Brain and GIPD_Brain data. 28,142 genes were overlapped. **(B)** The overlap genes of the GUB_Cage and GIPD_Cage data. There were 19,691 overlapped genes. **(C)** The overlap genes of the GUB_CD4 and GIPD_CD4 data. These two datasets have 5,656 identical genes. **(D)** The overlap genes of the GUB_CD8 and GIPD_CD8 data. These two datasets have 4,528 identical genes.

**FIGURE 3:**
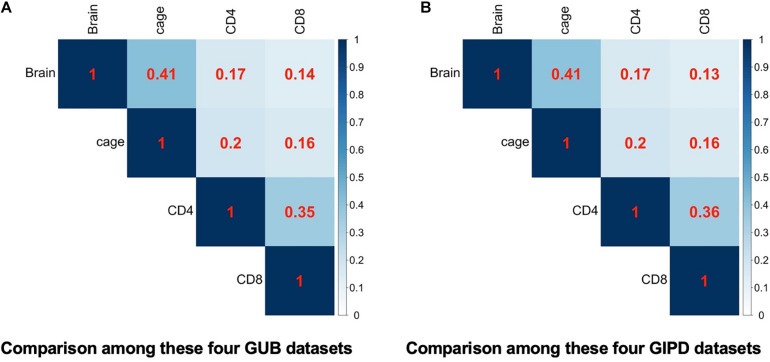
**(A)** The heatmap of the four GUB data. **(B)** The heatmap of the four GIPD data. The correlation of these four different eQTL datasets were compared under the background of the same GWAS dataset.

Subsequently, we compared the two datasets generated by using the same eQTL data and found that the genes overlapping between the two datasets accounted for about 98–100% of genes in each dataset, implying that the PD-associated genes identified in the two GWAS datasets are highly similar (Figure 2). Additionally, we analyzed the four datasets generated by the same GWAS dataset. This analysis showed that the overlap rate of the genes was not high among these datasets, with the largest overlap being less than 50% (Figure 3). Even though CD4 and CD8 are both markers of T lymphocytes, the gene overlap rate between the GUB_CD4 and GUB_CD8 datasets or between the GIPD_CD4 and GIPD_CD8 datasets were around 36%. This indicates a low degree of correlation between these eQTL datasets, which may be caused by the large differences in the number of SNPs found.

### SMR Analysis

The SMR approach was employed to analyze the eight datasets. The P_*SMR*_ value calculated was compared with the corresponding threshold value, and finally three risk genes were found (Tables 2, 3). As Supplementary Figure 3 shown, whether GUB GWAS or GIPD GWAS, the significant SNPs were only located on chromosome 4. However, the results of the SMR analysis showed that that GUB_Brain identified one target SNP locus associated with two genes: Mitochondrial Poly(A) Polymerase (MTPAP), RP11-305E6.4, which is located on chromosome 10. Coincidentally, GUB_Cage identified only this SNP locus, and the MTPAP gene associated with this SNP was also identified. Additionally, GIPD_Cage identified the highest number of the SNP sites of interest. These five SNPs were located on chromosome 4 and corresponded to the same Synuclein Alpha (SNCA) gene. However, the risk genes were not detected in the GIPD_Brain dataset. Similarly, based on Supplementary Figures 3E–H, no significant gene was identified for GUB _CD4, GUB _CD8, GIPD_ CD4, and GIPD_CD8 datasets, likely due to significantly lower *p*-values of CD4 and CD8 eQTL than those of the other two eQTL datasets. Thus, the SMR analysis of the 8 datasets identified a total of six candidate SNP loci and three genes, and the expression level of these three genes can affect the occurrence of PD.

**TABLE 2:** P_*SMR*_ threshold for these eight SMR datasets.

SMR dataset	GUB_Brain	GUB_Cage	GUB_CD4	GUB_CD8
P_*SMR*_ threshold	1.8×10^−6^	1.5×10^−6^	6.8×10^−6^	8.6×10^−6^
SMR dataset	GIPD_Brain	GIPD_Cage	GIPD_CD4	GIPD_CD8
P_*SMR*_ threshold	1.8×10^−6^	1.5×10^−6^	6.8×10^−6^	8.6×10^−6^

*The same eQTL datasets have the same threshold.*

**TABLE 3:** Discovery of PD causative gene by the Summary data-based Mendelian Randomization (SMR) analysis for these eight datasets.

SMR datasets	Number of discovered genes	Gene name	P value
GUB_Brain	2	MTPAP	7.215×10^−7^
		RP11-305E6.4	1.624×10^−6^
GUB_Cage	1	MTPAP	4.191×10^−7^
GUB_CD4	0	–	–
GUB_CD8	0	–	–
GIPD_Brain	0	–	–
GIPD_Cage	1	SNCA	4.671×10^−7^
GIPD_CD4	0	–	–
GIPD_CD8	0	–	–

*This list showed the number of genes found in different datasets, their names, and the value of P_*SMR*_ calculated.*

### Gene Function Analysis

Three candidate genes were SNCA, MTPAP, and RP11-305E6.4. To investigate how the expression of the three genes identified using SMR analysis contributes to the development of PD, we searched for their function in the KEGG database and related publications. The main pathological features of PD consist of the formation of Lewy bodies. The main component of the Lewy body is α-synuclein (α-syn) encoded by the SNCA gene. Mutation of this gene can cause the overexpression of α-syn, leading to the formation of Lewy bodies and hence the development of PD. The MTPAP gene encodes a nuclear polymerase responsible for generating homopolymerized (A) tails on mitochondrial mRNA. Although the search results did not reveal an evident relationship between this gene and PD, some studies show that mitochondrial dysfunction is involved in the pathogenesis of many neurodegenerative diseases, including PD. Abnormal mitochondrial structure or function has been found to induce a progressive loss of dopaminergic neurons and even trigger PD symptoms. Although the exact function of the polyadenylation of mitochondrial mRNA is unknown, the process is essential for maintaining correct mRNA expression in the mitochondria, and its disruption can lead to mitochondrial dysfunction. Therefore, the mutation of this gene may block the expression of MTPAP, causing mitochondrial dysfunction and leading to PD. The MTPAP gene is also known as PAPD1 or RP11-305E6.3, and another gene was found to be RP11-305E6.4. Both MTPAP and RP11-305E6.4 gene corresponds to the same SNP locus in this SMR analysis. This SNP may be localized in a non-coding gene regulatory region between these two adjacent genes. However, the function of this gene has not been identified yet, and its impact on PD remains unknown.

## Discussion

The SMR method was employed to integrate two GWAS datasets and four eQTL datasets. This approach identified six SNP candidate loci and three risk genes whose expression can significantly influence on the development of PD. The SNCA gene is the first confirmed pathogenic gene for PD (Lunati et al., 2018). It is located on chromosome 4 and contains six exons. The SNCA gene encodes the α-syn protein that is the main component of Lewy bodies (Mehra et al., 2019). α-syn is abundant in the brain and is also expressed in the heart, skeletal muscle, and other tissues. In the brain, α-syn is found primarily in presynaptic terminals, which release neurotransmitters essential for normal brain function. Mutation of the SNCA gene can cause the overexpression of the α-syn protein, leading to the formation of Lewy bodies and the development of PD. Different types of variations in the coding region and non-coding regions of the SNCA gene can increase its transcription and translation. The level of α-syn protein can also be increased by point mutation or copy number duplication of the SNCA gene (Kim, 2013). Moreover, in SNCA copy number repeat variation, the disease was more severe in the presence of the triploid type than the diploid type, indicating that the expression of α-syn may positively correlate with the severity of PD (Nussbaum, 2018). Therefore, the SNCA gene can be considered to be an effective target for the treatment of PD. SNCA is also believed to be involved in various other neurodegenerative diseases, such as Alzheimer’s disease, Lewy body disease, and muscular atrophy. Thus, the development of methods to inhibit SNCA gene mutations and decrease the formation of aggregates are of great clinical relevance.

Additionally, the polyadenylation of mRNA by the nuclear DNA-encoded mitochondrial poly(A) RNA polymerase is crucial for maintaining gene expression in human mitochondria (Lapkouski and Hällberg, 2015). Although the exact function of mitochondria mRNA transcription of adenosine acidification is not yet fully understood, the process is essential for ensuring correct mRNA expression in the mitochondria. MTPAP mutant proteins can shorten polyadenylation of mitochondrial mRNA, resulting in post-transcriptional downregulation of the expression of components of the respiratory chain complex and the impairment of an essential mitochondrial function (Wilson et al., 2014). Mitochondrial dysfunction is involved in many processes and diseases, including aging, cancer, diabetes, and neurodegenerative diseases such as PD and Alzheimer’s disease (Larsen et al., 2018). Among several mechanisms responsible for the pathogenesis of PD, mitochondrial dysfunction may be related to the death of dopaminergic neurons. Many of the PD-associated gene mutations result in an abnormal mitochondrial function and, eventually, neuronal damage, which is a critical component of the onset and development of the disease. Thus, compounds that target mitochondria and improve their function represent potential therapeutic options for delaying and treating degenerative diseases of the central nervous system.

RP11-305E6.4 is a long non-coding RNA (lncRNA) gene. LncRNAs are non-coding RNA molecules with a length of more than 200 nucleotides, which can govern gene expression, transcription, and post-transcription. Currently, there are few studies on this gene, and it is still unknown which genes are regulated by this lncRNA to influence the occurrence of PD. Nevertheless, the transcript of RP11-305E6.4 overlaps with that of MTPAP, and further studies could be conducted to determine whether this gene can regulate MTPAP to affect the occurrence of PD in the future.

In conclusion, we verified that the abnormal expression of the SNCA gene could lead to PD and found that the abnormal expression of the MTPAP and RP11-305E6.4 genes may also cause PD. This study further demonstrates that the design of drugs targeting SNCA gene is conducive to inhibit the formation of Lewy bodies, and to completely cure PD. Moreover, we have identified two new candidate genes for PD. This provides a research direction for understanding the biological significance behind the pathological features of PD.

## Data Availability Statement

Publicly available datasets were analyzed in this study. This data can be found here: http://ftp.ebi.ac.uk/pub/databases/gwas/summary_statistics/GCST007001-GCST008000/GCST007780/; http://ftp.ebi.ac.uk/pub/databases/gwas/summary_statistics/GCST010001-GCST011000/GCST010765/.

## Author Contributions

XC, CX, and LZ contributed to the design and implementation of the research, to the analysis of the results, and to the writing of the manuscript. All authors contributed to the article and approved the submitted version.

## Conflict of Interest

The authors declare that the research was conducted in the absence of any commercial or financial relationships that could be construed as a potential conflict of interest.

## Publisher’s Note

All claims expressed in this article are solely those of the authors and do not necessarily represent those of their affiliated organizations, or those of the publisher, the editors and the reviewers. Any product that may be evaluated in this article, or claim that may be made by its manufacturer, is not guaranteed or endorsed by the publisher.

## Supplementary Material

Supplementary Figure 1 **(A)** The Manhattan diagram of GUB GWAS data. **(B)** The Manhattan diagram of GIPD GWAS data.

Supplementary Figure 2 **(A)** The Manhattan diagram of Brain eQTL data. **(B)** The Manhattan diagram of Cage eQTL data. **(C)** The Manhattan diagram of CD4 eQTL data. **(D)** The Manhattan diagram of CD8 eQTL data.

Supplementary Figure 3 **(A)** The Summary data-based Mendelian Randomization (SMR) analysis results of GUB_Brain. **(B)** The SMR analysis results of GIPD_Brain. **(C)** The SMR analysis results of GUB_Cage. **(D)** The SMR analysis results of GIPD_Cage. **(E)** The SMR analysis results of GUB_CD4. **(F)** The SMR analysis results of GIPD_CD4. **(G)** The SMR analysis results of GUB_CD8. **(H)** The SMR analysis results of GIPD_CD8.
